# Challenges, priorities and tasks for the generalists at the time of the COVID-19 pandemic

**DOI:** 10.1080/13814788.2020.1791073

**Published:** 2020-07-17

**Authors:** Christos Lionis, Elena Petelos

**Affiliations:** Clinic of Social and Family Medicine, School of Medicine, University of Crete, Heraklion, Crete, Greece

Dear Editor,

We read with interest the Editorial of de Sutter and colleagues [1], highlighting coronavirus disease-2019 (COVID-19) challenges and implications for Family Medicine and Primary Care.

Therefore, we would like to share our reflections on issues of high relevance to Family Medicine and Primary Care, grouping them into key priority areas, i.e. research, healthcare policymaking and clinical practice.

## Research

A commonly used term, representing an intensely debated concept, is vulnerability. Most reports in the evidence base, including for observational studies conducted for COVID-19, utilise this term in reference to the frequency of co-existing chronic diseases, including diabetes mellitus and cardiovascular disease (CVD). Less attention has been given to the psychosocial factors, and the role they play to increase vulnerability or, conversely, to confer a protective effect, reducing COVID-19 severity and shortening its duration. Research conducted by the University of Crete has attempted to identify such determinants for chronic conditions, including for CVD [2]. Study design should allow correlation of exposures to protective factors, with data being harvested from electronic health records. Such type of evidence generation will contribute to evidence-based practice and shared decision-making.

## Healthcare policy

The role of Primary Care and Family Medicine to coordinate and support surveillance, including through community-based monitoring, has been discussed; however, concerted efforts are needed to improve the design and conduct of prevalence studies. The extend of the COVID-19 pandemic has decidedly demonstrated that the only way to monitor the pandemic effectively is to optimise the integration of public health and primary care. That implies sound communication channels and system-level interoperability to develop and execute preparedness plans [3]. There is, also, the definitive need for implementation research on integration, to match the call of the Astana Declaration [4], and to address the needs of multimorbid patients, including of those in long-term care, through people-centred care approaches.

## Clinical practice

For the four critical-for-quality domains, i.e. access, continuity, comprehensiveness, and coordination [5], the period of the lockdowns and the quarantine revealed a largely unaddressed need for well-organised home care services. The integration of technology could be a catalyst to build more comprehensive, readily accessible records and registries for the effective allocation of resources to these settings.

Individuals and families have been under enormous stress, experiencing fear and anxiety. To respond effectively, generalists have to be adept at using e-health tools and equipped with sound communication skills. Updating existing curricula to enhance the capacity of generalists with motivational interviewing and compassionate care skills should extend to self-compassion and self-protection, for a sustainable health workforce to increase the resilience of healthcare systems and communities alike. We have started sharing our experiences from the University of Crete, as we believe they are encouraging given lessons learned from austerity and the financial crisis [6]. Self-compassion skills are also crucial for informal caregivers to protect themselves; improving the level of literacy, and ensuring they are given timely access to the information and resources they need will further empower them.

Considering these challenges for Family Medicine and Primary Care, it is essential to exchange experiences, collaborating across disciplines and co-creating with patients, for evidence-informed policymaking, and for the people and for the society.
